# A Study on the Analysis of and Educational Solution for Digital Sex Crimes in Korea

**DOI:** 10.3390/ijerph20032450

**Published:** 2023-01-30

**Authors:** Woo-Chun Jun

**Affiliations:** Department of Computer Education, Seoul National University of Education, Seoul 06639, Republic of Korea; wocjun@snue.ac.kr; Tel.: +82-2-3475-2504

**Keywords:** digital sex crime, digital sex education, information and communication technology, information ethics, teenage crime

## Abstract

With the development and spread of information and communication technology, our society is experiencing side effects of digital culture while also benefiting from various digital cultures. Representative side effects have spread significantly, including Internet addiction, copyright infringement, personal information infringement, and digital sex crimes. Digital sex crimes are very serious crimes, and we must find their causes and strongly prevent and deal with them at the social level. In this study, the causes and routes of occurrence of digital sex crimes in Korea are analyzed using statistics on digital sex crimes at the national level over the past four years. The statistical analysis results are as follows. First, the main victims of digital sex crimes are women in their teens and twenties, though the number of male victims is steadily increasing. Second, illegal filming is the most common type of digital sex crime, but it is not statistically significant. In other words, various digital sex crimes are occurring evenly. Third, the relationship between the victim and the perpetrator demonstrates the most temporary relationship, and there is no significant correlation between direct and indirect recognition with respect to the route of crime recognition. Finally, deletion by a digital platform is the highest for adult sites compared to other platforms. Based on these analysis results, this study proposes educational countermeasures to digital sex crimes, such as the need for early education to prevent digital sex crimes and the diversification of crime-reporting methods via the establishment of an educational portal site.

## 1. Introduction

In the current knowledge- and information-based society, modern people enjoy various benefits of digital technology. Although these benefits make our lives rich and vibrant, digital technology has two sides; therefore, the side effects or adverse effects of digital technology use are also increasing. The representative side effects of digital technology are traditionally personal information infringement, hacking, copyright violation, and Internet addiction, though digital sex crimes have been increasing recently. Digital sex crimes are compared to murder in terms of their seriousness or impact. In other words, digital sex crimes are compared to murder because they insult an individual’s personality and body while causing irreparable humiliation. Specifically, digital sex crimes are referred to as character assassination and soul murder [1]. Character assassination is the act of intentionally and continuously damaging a person’s reputation and credibility. It is called character assassination because the damage caused by digital sex crimes continues throughout the victims’ lives. On the other hand, digital sex crimes can also be called soul murder because they cause victims to lose their identity and become unable to control their emotions. Therefore, digital sex crimes should be recognized very seriously in our society, and digital sex crimes should be thoroughly prevented and strongly dealt with.

The Nth Room incident was a representative digital sex crime and sexual exploitation case in Korea. From the second half of 2018 to March 2020, victims were lured using messenger apps such as Telegram. They were threatened to take part in sexual exploitation, then videos were distributed. The victims of this case included a large number of minors, including middle school students, and the number of participants in the crime was confirmed to be more than tens of thousands, including video holders and distributors [2,3,4].

Online sexual exploitation incidents, which have been known since late 2018 due to the Nth Room incident, have raised a social alarm regarding the risk of crimes and anonymous chat rooms that minors and adult women in their twenties are exposed to. Due to these incidents, digital sex crimes, such as digital sexual exploitation and online grooming, are often featured in the media and are widely known. Although social interest has increased, the risk of crime is increasing with the spread of digital and online cultures due to the rapid development of Internet technology and the influence of the COVID-19 pandemic. In particular, due to the COVID-19 pandemic, classes in elementary, middle, and high schools are not conducted face-to-face, and the risk of criminal damage for children and adolescents may increase as children and adolescents spend more time in front of computers without parental control. In fact, according to various statistics and the literature, rates of digital sex crimes are increasing, and crimes such as the production and distribution of sexual exploitation images of children and adolescents are increasing significantly [5,6]. As such, digital sex crimes are becoming more intelligent and organized. Recently, the production and distribution of digital sexual exploitation material using new information and communication technologies, such as the dark web, are increasingly expanding and evolving systematically. The damage is serious, semi-permanent, and highly scalable, raising the need for specific measures to block this damage and eradicate it [7,8].

Digital sex crimes are increasing day by day and their variety is diversifying. In order to properly and strictly cope with digital sex crimes, above all, the cause and process of digital sex crimes must be accurately identified. By accurately analyzing the causes and processes of digital sex crimes, digital sex crimes can be prevented in advance, and perpetrators should be dealt with severely for crimes that have already occurred. The purpose of this study is to accurately analyze the cause and process of digital sex crimes in Korea and present countermeasures. In order to achieve this purpose, this study analyzed the statistics of digital sex crimes at the national level over the past four years. This study suggests educational countermeasures based on the results of this statistical analysis.

The organization of this paper is as follows. First, Chapter Two introduces the definition and characteristics of digital sex crimes as related works and introduces previous works that present the current status of digital sex crimes in Korea. Chapter Three discusses the status and statistical analysis of digital sex crimes based on four years of statistics on digital sex crimes. Chapter Four introduces educational countermeasures and the cause analysis based on the digital sex crime statistical analysis. Finally, Chapter Five provides the conclusions of this study and addresses future research works.

## 2. Related Works

### 2.1. Definition of the Digital Sex Crime

As there is no stipulated, current law that defines the exact concept of what digital sex crimes are, digital sex crimes can be interpreted as policy terms rather than as legal terms in a strict sense. Various views are mixed, including the legal perspective of understanding these crimes as sex crimes and the feminist perspective of understanding them as gender-based violence. The terms “online sex crime”, “cyber sexual violence”, and “digital sex crime” are commonly used [9,10].

Recently, due to the nature of digital content and technology, sex crimes demonstrate aspects that can take place across offline and online spaces. Therefore, it is not reasonable to define them as “online sex crimes”. On the other hand, the concept of a “digital sex crime” can cover sex crimes that occur online and offline by reflecting changes in “online sex crimes”. On the other hand, “cyber sexual violence” is a concept that focuses on verbal sexual violence and the transmission of sexual images against the will of the other party. Recently, it has become difficult to limit it to the term “cyber sexual violence” because it shows sex crimes using various digital technologies such as deepfakes. According to Wikipedia, a deepfake (a hybrid of deep learning and fake) is a human image synthesis technology based on artificial intelligence. Using a machine learning technology called the generative adversarial neural network (GAN), existing photos or images are transformed by overlapping the original photos or images. Similarly, according to the Oxford dictionary, a deepfake is “a video of a person in which their appearance has been digitally altered so that they look like somebody else”.

According to a study in [7], a “digital sex crime” is comprehensively defined as “gender-based violence that occurs online and offline through digital devices and information and communication technology, and sexual violence that violates the sexual autonomy and personal rights of others in cyberspace”. Kim has suggested a similar definition of a digital sex crime [11]. Meanwhile, according to one study [10], “digital sex crimes” are comprehensively defined as “sex crimes occurring online and offline through digital devices and information and communication technology”. Specifically, they first include using a camera to film a person’s body without their consent; second, they involve the distribution, storage, exhibition, or threat to distribute the film without the other person’s consent; and third, they include sexual harassment, such as transmitting unwanted sexual images in media spaces such as SNS.

### 2.2. The Types and Characteristics of the Digital Sex Crime

Digital sex crimes can be mainstreamed in various types. First, in [7], the types of digital sex crimes were categorized into two main categories: online and online and offline crimes. In the online category, there were four subcategories: (1) threatening to use photographs made using cameras and sexual violence, (2) photo synthesis (deepfakes), (3) sexual harassment, and (4) digital grooming. These four subcategories are more specifically subdivided. First, “threatening to use photographs and sexual violence” can be divided into filming and distribution without the consent of the other party. Second, “photo synthesis (deepfake)” is the act of synthesizing and distributing a specific individual’s face or body image with sexual photos using face or voice synthesis technology. Third, “sexual harassment” is the act of transmitting sexual harassment and pornography without consent by inducing sexual harassment, the expression of insults, sexual jokes, or unwanted sexual conversations. Fourth, “digital grooming” refers to the act of approaching children and adolescents through online chats, mobile messenger services, and SNS to attract and tame victims, facilitate sexual exploitation, and prevent damage exposure. On the other hand, the online and offline category is a classification that demonstrates the relationship between other crimes occurring in real space and online sex crimes. Due to the nature of digital sex crimes, this is focused on dissemination and intimidation using images. The detailed subcategories were classified as (1) sexual coercion, (2) financial demand, (3) bullying, and (4) dating violence.

According to one study [11], the types of digital sex crimes can be classified into six categories. These include: (1) illegal filming: filming of the body, such as the inside of a skirt, the back, the whole body, the face, or the naked body without consent, or secretly filming the act of defecating or sexual activity; (2) distribution and re-distribution in a state of disagreement: uploading illegal filming materials on web hard, pornographic sites, SNS, etc., or distributing them in group chat rooms; (3) distribution and sharing: business operators and users who distribute illegally filmed materials using web hard, pornographic sites, SNS, etc. for commercial purposes, (4) threats to distribute: threats to distribute filmed materials to family members and acquaintances, threats to distribute filmed materials in a case of non-compliance, and demanding money by threatening to distribute the footage, (5) photo synthesis: photographs from the victim’s daily life are combined with sexual photos and then distributed, and (6) sexual harassment: an act of defaming or insulting a victim with sexual remarks online.

In [12], the types of digital sex crimes are divided into five types: (1) verbal harassment (sexual insults and demeans), (2) sending pornography and messages without consent, (3) image-use sexual violence (synthesis), (4) filming and distribution when there is disagreement over the activity, and (5) online grooming.

Table 1 summarizes the various types of digital sex crimes.

Combining the findings of various studies on digital sex crimes, the characteristics of digital sex crimes are as follows [11,13]. The damage of digital crimes tends to be underestimated due to the perception that they result in less damage than direct sex crimes. However, in the case of digital crimes, the victim’s wounds are more serious than in instances of ordinary sex crime victims. This because once sexual exploitation is leaked online, it spreads rapidly to an unspecified number of people. In other words, once pornography is distributed it is almost impossible to completely delete it, and the victim is damaged for a lifetime. Another characteristic of digital sex crimes is that, despite being a victim, there is a high possibility of social criticism or prejudice of the victim. In particular, if a film taken with the consent of the victim is illegally distributed, it is easy to form a false critical perception of the victim. In other words, it is difficult to say that the victim was unilaterally attacked by the perpetrator because the filming was spontaneous or consent-based; the reality is that the view that the victim is not responsible at all underlies our society [14].

### 2.3. Support for Victims of Digital Sex Crimes

The Women’s Human Rights Institute of Korea is in charge of supporting victims of digital sex crimes at the national level. According to the institute, damage support for digital sex crimes is supported in various ways. First, the institute provides support counseling on digital sex crimes and deleting digital sex crime contents. Second, it continuously monitors digital sex crime contents. Third, if investigation, law, and medical support are needed, related institutions are linked.

Meanwhile, financial support for victims of digital sex crimes is currently not being implemented. According to [15], the Ministry of Justice operates a victim support system that allows for financial support for general crime victims, but digital sex crime victims have very few support cases. In the case of digital sex crimes, support is difficult to obtain because the damage caused by the crime cannot be calculated. In 2019, digital sex crimes accounted for approximately 21% of the total violent crimes, but only 1.9% of the financial support was provided for victims of digital sex crimes [15].

### 2.4. Previous Works

Previous studies on the causes and routes of occurrence of digital sex crimes in Korea are as follows.

First, in [16], data from a national digital sex crime statistics database from 2018 to 2020 was analyzed to identify the causes of digital sex crimes in Korea. As a result of a statistical analysis, statistically significant conclusions were drawn. The first finding was that the main victims of digital sex crimes were women in their teens and twenties. Additionally, the typical types of digital sex crimes were the distribution of illegal contents and illegal filming, among various types of crimes. In addition, the perpetrators of digital sex crimes are mainly unknown, and digital sex crimes were less often recognized by others. This means that the victims recognize the crimes directly more often. Based on the statistical analysis results, various plans are proposed in opposition to digital sex crimes.

Meanwhile, in [17], the current status of digital sex crimes was investigated using the National Police Agency’s investigative database statistics, and measures to cope with digital sex crimes were proposed in terms of investigative agencies. Specifically, among digital sex crimes, crimes such as body cam phishing, obscene use of communication media, camera use, illegal video distribution, and child and youth sexual exploitation have been steadily increasing. Meanwhile, various countermeasures have been proposed to cope with digital sex crimes in this study.

In [18], the structure and characteristics of digital sex crimes against children and adolescents in Seoul were investigated. The results of the study are as follows. First, for children and adolescents, the online realm was a major place to create and expand social relationships. It was not uncommon for them to meet strangers through the Internet. The online world, which is believed to guarantee anonymity, is a playground for children and adolescents to express and practice their sexual curiosity. In addition, female children and adolescents experienced more frequent damage than male children and adolescents due to the damage characteristics, and middle and high school girls were more frequently affected by grade. On the other hand, various online and offline response environments for digital sex crimes against children and adolescents were proposed to respond to the digital sex crimes of Seoul teenagers, noting in particular the necessity of digital citizenship education for children and adolescents by gender and school level.

Meanwhile, in [19], Kim and Chun investigated the experience of sexual violence in the data relationships among digital sex crimes and suggested ways to support the victims. First, digital sexual violence in dating relationships consisted of continuous demands for filming by men and, unlike physical sexual violence, the damage continued to perpetuate due to the infinite distribution and spread of the videos. Additionally, people who recognized themselves and contacted them again realized that they were victims, and it has become difficult to recover from the damage of digital sexual violence over time. In order to respond to such sexual violence in dating, it is necessary to operate a campaign to prevent digital sexual violence and change social awareness. Additionally, active efforts by Internet companies are required to create a safe Internet environment. It was also suggested that an integrated support system for victims, such as the training of professional counselors. should be established.

## 3. Current Status and Analysis of Digital Sex Crimes in Korea

### 3.1. Current Status of the Digital Sex Crimes in Korea

This study first introduces the status of digital sex crimes in Korea. To introduce the most accurate and up-to-date status, national digital sex crime statistics provided by the Women’s Human Rights Institute of Korea (http://www.stop.or.kr, accessed on 12 December 2022) were used. The institute is a public institution, established in 2009 to professionally and systematically fulfill the nation’s responsibilities of preventing, protecting, and supporting victims of womens’ crimes. Meanwhile, in this study, the latest statistical data by the institute from 2018 to 2021 are cited, and an official report published by the institute is used to introduce the objective status [20]. In this paper, some of the digital sex crime statistical data are introduced.

First, Table 2 shows the current status of victims by year and age.

Meanwhile, Table 3 shows the types of digital sex crimes by year. In Table 3, “distribution” includes cases where sexual photographs taken with consent were distributed and cases where sexual photographs taken without consent were distributed. “distribution and threat” refers to cases of threatening to distribute sexual films. In addition, “distribution and anxiety” refers to cases of experiencing anxiety about the distribution of sexual films, and “cyberbullying” refers to cases of unwanted sexual harassment or transmission of sexual films.

Meanwhile, Table 4 shows the relationship between victims and perpetrators by year. In Table 4, a “temporary relationship” refers to a chat partner or a person who the victim only met once, and a “stranger” refers to a person who has been identified as the perpetrator but has no acquaintance with the victim. In addition, a “close relationship” refers to a spouse, ex-spouse, lover, or ex-lover, and a “social relationship” refers a person who was involved in work and social activities with the victim, such as at a school, workplace, or institution. Finally, “family relationship” refers to parents, brothers, and sisters, excluding spouses.

Table 5 shows the route of damage recognition by year. The route of damage recognition refers to how the victim perceives the digital sex crime committed against them. In other words, it means a path through which one can directly recognize the damage or others can first recognize it and inform the victim.

Table 6 shows the amount of deletion support by platform.

### 3.2. Analysis of Digital Sex Crimes

#### 3.2.1. Data

The data used in this study were based on the 2021 Digital Sex Crime Victim Support Report by the Women’s Human Rights Institute of Korea [20]. This report is a digital sex crime status report published annually since 2018, and the data used in this study do not require the consent of survey respondents because it is secondary data used by citing the report in [20].

#### 3.2.2. Methodology

The data in this study were analyzed using the SPSS (Statistical Package for the Social Science) WIN 27.0 program. As an analysis method, an ANOVA (variable analysis) and a *t*-test (verification) were performed to analyze the statistical data. In addition, a Scheffe test was conducted as a post hoc verification.

#### 3.2.3. Analysis

In this study, the following six hypotheses are first established to accurately and meaningfully analyze the digital sex crime status data introduced in Section 3.1.

(1) The main victims of digital sex crimes are women in their teens and twenties;
(2) The number of male victims is constantly increasing;
(3) Among the types of crime, illegal filming is the most common;
(4) In the relationship between the victim and the perpetrator, most are temporary relationships, though some are unknown;
(5) In the route of damage recognition, victim’s direct recognition is increasing;
(6) Delete support is the most common for adult sites.

(1) Victims of digital sex crime by age for women

Table 7 shows the results of examining digital sex crime according to women’s age. a and b represent the results of the post-hoc test, respectively.

According to the age of the women, the average of those in their twenties was the highest, followed by those in their teens, those in their thirties, and those in their fifties or older. There was a statistically significant difference (F = 4.22, *p* < 0.05). In addition, as a result of the post hoc verification, there was a significant difference between the age groups of the tens and forties, tens and fifties, twenties and forties, and the twenties and over-fifties. Therefore, female victims of digital sex crimes are more often in their teens and twenties than other age groups, thus establishing Hypothesis 1.

(2) Male victims of digital sex crimes by year

Table 8 shows the results of examining the trend of digital sex crime damage in men by year. a and b represent the results of the post-hoc test, respectively.

Regarding the trend of male victims of digital sex crimes by year, the average was higher in the order of 2018, 2019, 2020, and 2021, and there was a statistically significant difference (F = 5.63, *p* < 0.01). In addition, as a result of the post hoc verification, a significant difference was between 2018 and 2021 and 2019 and 2021. Therefore, digital sex crimes perpetrated on men are increasing, and Hypothesis 2 is established.

(3) Analysis of crime type

The results of examining the types of digital sex crimes are shown in Table 9.

In terms of the type of digital sex crimes, the illegal filming had the highest average, followed by distribution, distribution and anxiety, Distribution and threat, and cyberbullying. the average of photo synthesis was the lowest; however, there was no statistically significant difference. Therefore, digital sex crimes do not differ much by type of damage and Hypothesis 3 is rejected.

(4) Relationship between victims and perpetrators

Table 10 shows the results of examining the relationship between the victims and perpetrators. a and b represent the results of the post-hoc test, respectively.

Looking at the relationships between victims and perpetrators, the average of temporary relationships was the highest, followed by close relationships, strangers, and social relationships, with family relationships were having the lowest average, showing statistically significant differences (F = 3.26, *p* < 0.05). In addition, as a result of post hoc verification, temporary relationship and stranger, temporary relationship and social relationship, and temporary relationship and family relationship showed significant differences. Therefore, victims of digital sex crimes most commonly have a temporary relationship with the perpetrator have a family relationship with the perpetrator in the least number of instances. Therefore, Hypothesis 4 is established.

(5) Route of damage recognition

Table 11 shows the route of damage recognition.

As shown in Table 11, the results of examining the route of damage recognition of digital sex crimes showed that there was no significant difference between direct recognition and recognition by others. Thus, Hypothesis 5 is rejected.

(6) Deletion support by platforms

Table 12 shows the results of examining the number of deletion support by platforms. a and b represent the results of the post-hoc test, respectively.

Looking at the deletion support by platform, the average of adult sites was the highest, followed by social media, search engine, community, and P2. The average of others was the lowest, and there was a statistically significant difference (F = 2.88, *p* < 0.05). In addition, as a result of post hoc verification, adult site and other, social media and other, and search engine and other showed significant differences. Therefore, digital sex crime deletion support is the most frequently provided on adult sites among platforms, and Hypothesis 6 is established.

#### 3.2.4. Result

From the above analysis, we have the following results on whether the six hypotheses are established/rejected.

- Hypothesis 1: Established
- Hypothesis 2: Established
- Hypothesis 3: Rejected
- Hypothesis 4: Established
- Hypothesis 5: Rejected
- Hypothesis 6: Established

## 4. Discussion

### 4.1. Analysis of Digital Sex Crimes

Based on the statistical analysis results of digital sex crimes analyzed in Chapter Three, the following discussions and interpretations can be made.

First, the first hypothesis that the main victims of digital sex crimes were women in their teens and twenties was established. In more detail, teenagers and people in their twenties suffer relatively more damage from digital sex crimes. For people in their thirties, the damage suffered can be seen as moderate overall, and those in their forties and fifties or older experienced relatively little damage. The analysis that many victims of digital sex crimes are women, especially young women, is consistent with previous studies [15,21,22]. In particular, it is worth noting that the age of damage from digital sex crimes (that is, the victim’s age of damage) is gradually decreasing [21]. As can be seen from the establishment of Hypothesis 2, the number of male victims is also increasing. In the case of male victims of digital sex crimes in particular, the number of victims has been rapidly increasing in recent years. The increase in male victims cannot necessarily be concluded to be due to an increase in female perpetrators. In other words, there may be same-sex offenders. According to a recent report [23], the cause of the surge in male victims of digital sex crimes is illegal filming threats. On the other hand, the increase in digital sex crimes is due to the increase in the use of electronic devices such as smartphones, but it can also be interpreted as an increase in the reporting rate due to changes in social perception [20]. In addition, digital sex crimes are more likely to have victims or perpetrators in lower age groups than traditional sexual violence crimes. Recently, the number of cases targeting adults has been decreasing, but the number of cases in which children and adolescents are victims and perpetrators continues to increase [21].

On the other hand, the rejection of Hypothesis 3 means that illegal filming accounts for a large proportion of all digital sex crimes but is not statistically significant. Therefore, various types of digital sex crimes are occurring, and digital sex crimes are diversifying due to the development of technology, and crimes are organically related to each other. For example, the increase in illegal filming naturally results in an increase in distribution. On the other hand, it can be concluded that the establishment of Hypothesis 4 has the most temporary relationship between the victim and the perpetrator. In particular, regarding the relationship between the victim and the perpetrator, the temporary relationship occupies an overwhelming proportion of cases when compared to other relationships. Since a temporary relationship is not a close relationship, the perpetrator can treat the victim carelessly, and it can be seen as a relationship in which the perpetrator can easily hide his or her identity or trace.

In addition, the rejection of Hypothesis 5 means that there is no difference between direct and indirect recognition (recognition by others) in terms of the route of crime recognition. In other words, the victim may recognize the crime by identifying it themselves, or others may identify it first. Ultimately, it is not important who recognizes the crime first because the timing of crime recognition is accelerated. Finally, the establishment of Hypothesis 6 means that the place in which digital sex crimes occur most actively are ultimately the most adult sites. In a broader sense, it can be seen that deletion support occurs more for adult sites, social media, and search engines than other platforms. Therefore, controlling and managing access to adult sites can reduce digital sex crimes.

### 4.2. Educational Solutions to Digital Sex Crimes

As previously argued in [21], digital sexual violence crimes are characterized by the low age of the suspect and also the use of mobile phones to commit crimes against unspecified persons who cannot be identified. The age of the victims and perpetrators of digital sex crimes continues to decrease because teenagers can easily access pornography using popularized smartphones while they are not yet sensible and have distorted adult awareness. Therefore, it is very necessary to develop the right perception of sex from an early age and to provide effective education on gender sensitivity.

The best way to deal with digital sex crimes is to provide preventive education for young adolescents. In other words, no one can deny that prevention is a very good method, rather than punishment. However, there are no official subjects or curricula for digital sex education in elementary, middle, and high schools in Korea. The inclusion of digital sex education in public education is expected to take a considerable amount of time because it requires various discussions and formal processes. Therefore, the following method is needed as an educational method to prevent and cope with the rapidly increasing number of digital sex crimes. The most urgent task is to establish an educational portal site to prevent and deal with digital sex crimes. This portal site should provide various educational materials, including videos, and also provide online counseling functions for teenagers to consult at any time, so that they can receive counseling at any time through phone calls or online bulletin boards. In addition, it is necessary to operate a hotline that allows young victims to report crimes immediately and provides the function to receive immediate protection along with reporting.

## 5. Conclusions

Due to the rapid development of digital technology and the spread of digital culture, modern people are enjoying various benefits and also experiencing various side effects of technology. Among these side effects, the most serious one can be digital sex crimes. This is because digital sex crimes are characterized by the fact that perpetrators can easily commit crimes against many people, while victims live in pain for life. Currently, rates of digital sex crimes are steadily increasing in Korea, and crime types are gradually diversifying. Therefore, it is necessary to accurately analyze the current status of digital sex crimes and prepare countermeasures. To this end, this study analyzed the statistics of digital sex crimes over the past four years.

As a result of this study, we identified the following findings. First, victims are women in their teens and twenties, and the number of male victims is increasing. In addition, there are no statistically significant crimes among the types of digital sex crimes. In addition, the relationship between the victim and the perpetrator is most often a temporary relationship, and there is no significant correlation between direct and indirect recognition in terms of crime recognition. In addition, deletion by the digital platform hosting the material is significantly higher for adult sites than for other sites. Based on these results, this study proposes the establishment of an educational portal site for the digital sex education of adolescents.

Meanwhile, the punishment for digital platforms related to digital sex crimes should also be strengthened. In the case of digital sex crimes, it may occur as an individual-to-individual interaction between the perpetrator and the victim, or it may occur as an individual-to-group, or a group-to-group interaction through a digital platform, such as in the case of the Nth Room incident mentioned in the introduction. In this case, digital platforms should also be punished because they serve as a medium or relay for the crimes. However, in Korea, most digital platforms that distribute pornography or digital sexual exploitation content related to digital sex crimes have servers overseas, especially in underdeveloped countries. Therefore, it is very difficult to find digital platform managers and it is very complicated to prosecute, punish, and obtain financial compensation for victims under international law due to the requirement of various processes and procedures. The detection and punishment of digital platforms operating with servers overseas is not currently easy, and it is expected to take some time because international cooperation is needed.

Meanwhile, digital sex crimes can be viewed as gender-based violence in a broad sense. Therefore, an understanding of gender-based violence is needed. According to UNHCR (The UN Refugee Agency), gender-based violence refers to harmful acts directed at an individual based on their gender. It is rooted in gender inequality, the abuse of power, and harmful norms. There are many diverse causes of gender-based violence, including social, cultural, and political factors. In order to prevent and properly cope with digital sex crimes, the fundamental causes should be studied along with a comprehensive understanding of gender-based violence in the future.

In this study, educational countermeasures were proposed as fundamental measures against digital sex crimes. Educational countermeasures are a very fundamental method and can be particularly effective in crime prevention. However, educational countermeasures have limitations in responding to digital sex crimes that use rapidly developing technologies, and there are limitations that cannot be applied to teenagers who do not receive public education. Therefore, educational countermeasures should be implemented not only at school but also at home. In other words, there is a limit in time and space to guiding students in school. Therefore, educational countermeasures must include home education.

The future research of this study are as follows. First, in order to analyze the causes of digital sex crimes in more depth, a psychological analysis using interview data or counseling data should be necessary. This psychological analysis can provide more fundamental measures to prevent digital sex crimes. In addition, it is necessary to develop various educational materials for educating youth about digital sex crimes. In particular, it is essential to develop timely and interesting educational contents suitable for an adolescent level.

## Figures and Tables

**Table 1:** Summarization of the Various Types of Digital Sex Crimes.

Reference	Types of Digital Sex Crime
Kim and Park [7]	Online Type: (1) Threatening to use photographs using cameras and sexual violence (2) Photo synthesis (3) Sexual harassment (4) Digital grooming
	Online and Offline Type: (1) Sexual coercion (2) Financial demand (3) Bullying (4) Dating violence
Kim [11]	(1) Illegal filming (2) Distribution and re-distribution in a state of disagreement (3) Distribution and sharing (4) Threats to distribute (5) Photo synthesis (6) Sexual harassment
Kim et al. [12]	(1) Verbal harassment (2) Sending pornography and messages without consent (3) Image-use sexual violence (4) Filming and distribution of disagreement (5) Online grooming

**Table 2:** Status of Victims by Year and Age.

Year	Gender	10s	20s	30s	40s	Over 50	Unknown
2018	Female	95	218	98	18	20	657
	Male	16	33	11	16	5	128
2019	Female	288	463	148	33	22	878
	Male	33	41	19	17	10	135
2020	Female	1007	863	267	77	36	1797
	Male	197	189	65	57	51	367
2021	Female	1194	1090	367	91	42	2325
	Male	287	371	104	81	96	904

Unit: number of people.

**Table 3:** Types of Digital Sex Crimes by Year.

Year	Illegal Filming	Photo Synthesis	Distribution	Distribution and Threat	Distribution and Anxiety	Cyber-Bullying	Unknown
2018	656	69	758	208	216	108	274
2019	1043	144	1213	354	557	274	530
2020	2239	349	1586	978	1050	306	486
2021	2228	176	2103	1939	2660	533	714

Unit: number of occurrences. Duplicated responses allowed.

**Table 4:** Relationship between victims and perpetrators by year.

Year	Temporary Relationship	Stranger	Close Relationship	Social Relationship	Family Relationship	Unknown
2018	198	169	309	136	5	498
2019	331	373	500	227	5	651
2020	1239	280	429	227	15	2783
2021	1963	548	539	290	17	3595

Unit: Number of people.

**Table 5:** Route of Damage Recognition by Year.

Year	Direct Recognition	Recognition by Others	Unknown
2018	74	103	64
2019	200	187	138
2020	477	817	514
2021	964	560	1265

Unit: number of people.

**Table 6:** Deletion support by platform.

Year	Adult Site	Social Media	Search Engine	Community	P2P	Web Hard	Other
2018	8239	10,312	6705	848	2158	317	300
2019	26,170	4337	31,369	2042	29,358	190	1616
2020	38,332	65,894	25,383	14,550	5152	45	9494
2021	59,113	31,980	30,372	29,608	5181	344	13,222

Unit: Number of occurrences.

**Table 7:** Digital Sex Crimes by Age for Women.

Age	Mean	SD	F	*p*	Scheffe
10s a	646.00	536.16	4.22 *	0.017	a > b
20s a	658.00	391.69			
30s ab	220.00	120.95			
40s b	54.75	34.80			
Over 50 b	30.00	10.71			

* *p <* 0.05.

**Table 8:** Status of male victims of digital sex crimes by year.

Year	Mean	SD	F	*p*	Scheffe
2018 a	16.20	10.43	5.63 **	0.008	a < b
2019 a	24.00	12.65			
2020 ab	111.80	74.35			
2021 b	187.80	132.53			

** *p <* 0.01.

**Table 9:** Type of Digital Sex Crime.

Type	Mean	SD	F	*p*
Illegal Filming	1541.50	814.53	2.71	0.054
Photo Synthesis	184.50	118.48		
Distribution	1415.00	570.10		
Distribution and Threat	869.75	787.17		
Distribution and Anxiety	1120.75	1081.77		
Cyberbullying	305.00	174.95		

**Table 10:** Relationship between victims and perpetrators.

Relationship	Mean	SD	F	*p*	Scheffe
Temporary Relationship a	932.75	828.08	3.26 *	0.041	a > b
Stranger b	342.50	160.38			
Close Relationship ab	444.25	101.01			
Social Relationship b	220.00	63.39			
Family Relationship b	10.50	6.40			

* *p <* 0.05.

**Table 11:** Route of Damage Recognition.

Type	Mean	SD	t	*p*
Direct Recognition	428.75	394.54	0.05	0.964
Recognition by Others	416.75	332.64		

**Table 12:** Deletion Support number by Platforms.

Platform	Mean	SD	F	*p*	Scheffe
Adult Site a	32,963.50	21,370.30	2.88 *	0.038	a > b
Social Media a	28,130.75	27,836.01			
Search Engine a	23,457.25	11,471.06			
Community	11,762.00	13,414,50			
P2P	10,462.50	12,677.25			
Other b	3191.00	5161.06			

* *p <* 0.05.

## Data Availability

Publicly available datasets were analyzed in this study. This data can be found here: (http://www.stop.or.kr) (accessed on 12 December 2022).
